# Impact of the COVID-19 Pandemic on People Living With Rare Diseases and Their Families: Results of a National Survey

**DOI:** 10.2196/48430

**Published:** 2024-02-14

**Authors:** Maurizio Macaluso, Marc E Rothenberg, Thomas Ferkol, Pierce Kuhnell, Henry J Kaminski, David W Kimberlin, Michael Benatar, Mirna Chehade, The Principal Investigators of the Rare Diseases Clinical Research Network – Cycle 4

**Affiliations:** 1 Division of Biostatistics and Epidemiology Cincinnati Children's Hospital Medical Center Cincinnati, OH United States; 2 Department of Pediatrics University of Cincinnati College of Medicine Cincinnati, OH United States; 3 Division of Allergy and Immunology Cincinnati Children's Hospital Medical Center Cincinnati, OH United States; 4 Department of Pediatrics University of North Carolina at Chapel Hill Chapel Hill, NC United States; 5 Department of Neurology and Rehabilitation Medicine George Washington University Washington, DC United States; 6 Division of Pediatric Infectious Diseases University of Alabama at Birmingham Birmingham, AL United States; 7 Department of Neurology University of Miami Miami, FL United States; 8 Mount Sinai Center for Eosinophilic Disorders Departments of Pediatrics and Medicine Icahn School of Medicine at Mount Sinai New York, NY United States; 9 See Acknowledgments

**Keywords:** rare diseases, rare, chronic, COVID-19 infection, cross-sectional survey, access to care, changes in symptoms and use of medications, psychological impact on self and family, access, accessibility, cross-sectional, national, nationwide, survey, surveys, COVID-19, SARS-CoV-2, coronavirus, comorbid, comorbidity, vulnerable

## Abstract

**Background:**

With more than 103 million cases and 1.1 million deaths, the COVID-19 pandemic has had devastating consequences for the health system and the well-being of the entire US population. The Rare Diseases Clinical Research Network funded by the National Institutes of Health was strategically positioned to study the impact of the pandemic on the large, vulnerable population of people living with rare diseases (RDs).

**Objective:**

This study was designed to describe the characteristics of COVID-19 in the RD population, determine whether patient subgroups experienced increased occurrence or severity of infection and whether the pandemic changed RD symptoms and treatment, and understand the broader impact on respondents and their families.

**Methods:**

US residents who had an RD and were <90 years old completed a web-based survey investigating self-reported COVID-19 infection, pandemic-related changes in RD symptoms and medications, access to care, and psychological impact on self and family. We estimated the incidence of self-reported COVID-19 and compared it with that in the US population; evaluated the frequency of COVID-19 symptoms according to self-reported infection; assessed infection duration, complications and need for hospitalization; assessed the influence of the COVID-19 pandemic on RD symptoms and treatment, and whether the pandemic influenced access to care, special food and nutrition, or demand for professional psychological assistance.

**Results:**

Between May 2, 2020, and December 15, 2020, in total, 3413 individuals completed the survey. Most were female (2212/3413, 64.81%), White (3038/3413, 89.01%), and aged ≥25 years (2646/3413, 77.53%). Overall, 80.6% (2751/3413) did not acquire COVID-19, 2.08% (71/3413) acquired it, and 16.58% (566/3413) did not know. Self-reported cases represented an annual incidence rate of 2.2% (95% CI 1.7%-2.8%). COVID-19 cases were more than twice the expected (71 vs 30.3; *P*<.001). COVID-19 was associated with specific symptoms (loss of taste: odds ratio [OR] 38.9, 95% CI 22.4-67.6, loss of smell: OR 30.6, 95% CI 17.7-53.1) and multiple symptoms (>9 symptoms vs none: OR 82.5, 95% CI 29-234 and 5-9: OR 44.8, 95% CI 18.7-107). Median symptom duration was 16 (IQR 9-30) days. Hospitalization (7/71, 10%) and ventilator support (4/71, 6%) were uncommon. Respondents who acquired COVID-19 reported increased occurrence and severity of RD symptoms and use or dosage of select medications; those who did not acquire COVID-19 reported decreased occurrence and severity of RD symptoms and use of medications; those who did not know had an intermediate pattern. The pandemic made it difficult to access care, receive treatment, get hospitalized, and caused mood changes for respondents and their families.

**Conclusions:**

Self-reported COVID-19 was more frequent than expected and was associated with increased prevalence and severity of RD symptoms and greater use of medications. The pandemic negatively affected access to care and caused mood changes in the respondents and family members. Continued surveillance is necessary.

## Introduction

### Background

The COVID-19 pandemic, caused by the SARS-CoV-2, emerged in the fall of 2019 in Wuhan, China, and rapidly spread throughout the world. In March 2020, the World Health Organization (WHO) declared the outbreak to be a pandemic, and as of August 30, 2023, the WHO has reported >770 million confirmed cases and >6.9 million confirmed deaths attributable to the disease [1,2]. More than 103 million cases and 1.1 million deaths from COVID-19 have occurred in the United States [1,3], with devastating consequences for the health system [4,5] and the well-being of the entire population [6].

The *Journal of Medical Internet Research* and affiliated *JMIR* publications, including *JMIR Public Health and Surveillance*, have extensively documented the global impact of the COVID-19 pandemic; a total of 99 articles have reported the results of web-based surveys and analyses of internet browsing patterns and social media postings (search terms “Rare disease” and “COVID-19,” run on July 7, 2023). For example, during the early months of the pandemic, an artificial intelligence–based analysis of >902,000 tweets was used to identify sentiments regarding COVID-19 in the United States [7], and large-scale web-based surveys were conducted in China [8,9], South Korea [10], Russia [11], and Spain [12]. Several surveys have documented a negative impact on anxiety and depression, self-reported well-being, and quality of life in the general population [11-16] and in vulnerable groups such as pregnant women [17-19], university students [20-22], older people [23,24], and health care workers [25,26].

Despite the extensive *JMIR* literature on the impact of the pandemic, only a single study described the experience of people living with rare diseases (RDs), which documented the impact of the pandemic on conversation patterns in the Reddit platform (subreddit r/CysticFibrosis) [27]. Indeed, the literature on the impact of the pandemic on people living with RDs is sparse even 3 years into the pandemic. A PubMed search (search terms “Rare disease” and “COVID-19,” run on July 6, 2023) identified 148 potential matches, but review of the abstracts indicated that most publications were case reports, discussion papers, or other reports that do not entail data collection from people living with RDs: only 25 articles reported results of cross-sectional surveys or longitudinal studies of patients with RD or their caregivers [28-52]. Large studies (≥50 participants) in Asia [29,37,40], Europe and the United Kingdom [30,33,36,41,49,51], the United States [45,52], and Brazil [43] reported variable impact of the pandemic, including increased risk of acquiring COVID-19 [33,37,39], increased COVID-19–related mortality [33,37], increased anxiety and depression and other psychological problems [29,30,36,41,51,52], low quality of life [41,51], difficulties with access to care [36,40,43,45,52], and difficulties complying with public health recommendations such as masking [49].

According to US laws, an RD is defined as a condition affecting <200,000 individuals at a given time [53,54]. The Genetic and Rare Disease information system of the National Center for Advancing Translational Sciences lists >10,000 RDs [55,56]. Although each condition is rare, the aggregate prevalence may amount to 30 million [55]. RDs are often difficult to diagnose [57,58], most often lack specific treatments [59], and place a significant burden on families and society [60]. Some people with RDs have primary respiratory manifestations [61], immunocompromised states [62], and chronic comorbidities including intellectual disabilities [63]. People with RDs report a lower quality of life than the general population [64]; they are vulnerable and dependent on the health care system [65].

The Rare Diseases Clinical Research Network (RDCRN) is funded by 10 of the National Institutes of Health (NIH) to promote progress in the diagnosis and treatment of RDs. The RDCRN comprises 20 Rare Disease Clinical Research Consortia and a Data Management and Coordinating Center, >200 research sites worldwide, and many patient advocacy groups that ensure that patients’ voices are heard. The RDCRN has been active for 2 decades, and >40,000 people have participated in its studies [66].

### Objectives

The RDCRN was strategically well placed to study the impact of the pandemic among people living with RDs in the United States. We designed a cross-sectional survey to describe the characteristics of the COVID-19 infection in this population, to determine whether patient subgroups were affected more frequently or experienced increased severity, to determine whether the pandemic changed RD symptom and treatment patterns, and to understand the broader impact of the pandemic on people living with an RD and their families.

## Methods

### Overview

We designed a cross-sectional survey to describe the characteristics of the COVID-19 infection in the population with RD. People who lived in the United States, had an RD, and were aged <90 years (including children of any age) were eligible for the survey. There were no explicit exclusion criteria beyond failure to meet the eligibility criteria. The survey was advertised through the internet, social media, an NIH press release, and outreach by patient advocacy groups. Interested individuals were asked to access a dedicated web page and complete a REDCap (Research Electronic Data Capture; Vanderbilt University [67]) survey instrument on the web (Multimedia Appendix 1).

The data collection instrument was designed ad hoc by the authors in spring 2020, with contributions from all the principal investigators of the RDCRN consortia (included as a group author as “The Principal Investigators of the Rare Diseases Clinical Research Network - Cycle 4” refer to the collaborators list), based on their expert knowledge of RDs. The instrument comprises 8 sections:

- Section 1 includes eligibility and preliminary information criteria on whether the patient or a surrogate is interested in participating, whether the target respondent lives in the United States and has an RD, and whether the respondent has participated in RDCRN research;
- Section 2 explains the study and asks for consent offering three options: (1) respond to the survey, provide contact information, and allow contact for follow-up and opportunities for participating in RD research; (2) allow linkage of the survey results with data about the same individual provided as part of RDCRN studies; and (3) respond to the survey without any identifiers (one-time, anonymous response);
- Section 3 is restricted to individuals who agree to provide identifiers and includes name, date of birth, address, telephone number, and email address to allow future contacts;
- Section 4 includes sociodemographic variables (state of residence, age, sex, race and ethnicity), RD diagnosis including symptoms and comorbidities during the months preceding the COVID-19 epidemic in the United States, treatments received during the same period, smoking habits, and use of tetrahydrocannabinol-containing products and psychoactive drugs. It also includes questions about COVID-19–related symptoms that the respondent may have experienced before the beginning of the pandemic and any changes in RD symptoms and treatment experienced after the beginning of the pandemic in the United States (March 2020);
- Section 5 ascertains whether the respondent had a positive COVID-19 test or a clinical diagnosis of COVID-19 respiratory syndrome; and
- Sections 6 to 8 separately address respondents who did not acquire COVID-19 (section 6), acquired it (section 7), or did not know (section 8).

All these sections include a checklist of symptoms compatible with COVID-19; ask whether the pandemic interfered with access to medical care, RD treatment, and special food related to RD management; whether stay-at-home orders affected their mood or behavior; and whether the respondent or family members had to seek professional help to cope with stress and anxiety. A final question asks if the target respondent died during the interval leading to the survey. In addition to the abovementioned common questions, section 7 asks additional questions about the course of the COVID-19 infection, the need for hospitalization and special treatment (assisted ventilation), the duration of the illness, and whether the respondent received investigational drugs to treat COVID-19.

### Study Size Considerations

We aimed at enrolling 5000 participants based on a precision analysis, rather than a power analysis, because the survey was not designed to test specific hypotheses. At the time of planning the survey, the WHO estimates suggested that the prevalence of COVID-19 infection may be between 1% and 2%. Thus, if the patients with RD experienced the same risk as the general population, the target sample size would provide information on approximately 50-100 COVID-19 cases. As the 95% CI of 50 observed events was 37-66 based on the Poisson distribution, our estimate of the overall prevalence of COVID-19 infection among respondents would be very precise. The precision of survey measures of more common characteristics and experiences among respondents (eg, changes in access to treatment) would be much higher. Furthermore, given a sample size of 50 cases, the 95% CI of a simple mean would be between +0.25 and –0.25 SD units from the mean, affording adequate precision of measures such as the average duration of hospitalization, and estimates of rates for any categorization of the case group into 5-10 subgroups would also yield adequate precision in describing the variation in risk across population subgroups.

### Ethical Considerations

As the survey was conducted on the web, the REDCap survey instrument verified the eligibility of a prospective participant, provided an explanation of the objectives and process of data collection, verified consent, and gathered the required information. First, the instrument assessed the eligibility of the intended participant (ie, the person was living with an RD, was living in the United States, and was aged <90 years), automatically terminating data collection from ineligible individuals; ascertained whether the intended participant or a surrogate would be completing the survey; and ascertained whether the potential participant had participated in the RDCRN research. Next, it provided a comprehensive explanation of the study objectives and process and asked whether (1) the participant agreed to respond to the survey, provide contact information, and allow future contact for follow-up interviews and opportunities to participate in RD research; (2) if agreeing to (1), the participant further agreed to allow linkage of the survey results with data about the same individual provided as part of the RDCRN studies; or (3) the participant agreed to respond to the survey without any identifiers (1-time, anonymous response). The explanatory language clarified that no adverse events were expected except for a possible breach of confidentiality for participants who agreed to provide identifiable information and that the study team intended to minimize the risk of a confidentiality breach by keeping identifiers and survey responses in separate files and by presenting the study results in a format that would not allow the reidentification of individual patients with RD. No compensation was offered for participating in this study. The institutional review board (IRB) at Cincinnati Children’s Hospital Medical Center reviewed and approved the research protocol, all recruitment materials, and the survey instrument, including the language explaining the survey and asking for consent. The IRB granted a waiver of documentation of informed consent (IRB ID 2020-0299).

### Data Analysis

In this report, we aimed to address the following outstanding questions in the field:

1. How frequent was the self-reported laboratory or clinical diagnosis of COVID-19 among people living with RDs? Did this differ from the expectation based on population rates?
2. How frequent were COVID-19–related symptoms according to self-reported COVID-19 status?
3. What were the characteristics of COVID-19 infection (duration of the infection, need for hospital admission, and complications of the infection among COVID-19 cases)?
4. Did COVID-19 status influence changes in the frequency and intensity of RD-related symptoms and in the frequency of RD-related treatment?
5. Did the COVID-19 epidemic change access to care, access to special food and nutrition, demand for professional assistance to address stress and coping among people who live with RD, and was the impact affected by COVID-19 status?

We used simple descriptive statistics (frequencies and percentages for count variables and means, medians, and ranges for continuous variables) and provided 95% CIs as a measure of the precision of the estimates for survey results. Statistical hypothesis testing was used for select analyses.

To identify participants who acquired COVID-19, we used the answer to the simple survey question, “Did you acquire COVID-19?” which allowed the answers yes, no, or do not know (Multimedia Appendix 1, survey item 5.1). The 3-category answer was a pragmatic approach to identifying cases of infection because at the time we conducted the survey, access to laboratory tests remained limited.

We estimated the denominator at risk of infection considering each respondent at risk every day from the beginning of the pandemic to the day they reported acquiring COVID-19 (the outcome event) or the day they filled out the survey, whichever was earlier, as is done in retrospective cohort studies. We computed exact 95% CIs for the number of cases and the corresponding incidence rate under the assumption that the number of cases follows the Poisson distribution. We compared the monthly number of self-reported COVID-19 cases with the number expected based on data reported by the New York Times [68], computed by multiplying the national monthly infection rate by the number of respondents who were at risk of acquiring a COVID-19 infection during that month. The cases reported in the New York Times database are a mix of laboratory-confirmed cases and cases meeting the state-determined diagnostic criteria. We present the observed and expected cases in a graph and test the null hypothesis that the number of observed cases equals the expected value using an exact Poisson distribution method.

For all respondents before the beginning of the pandemic (Multimedia Appendix 1, survey instrument item 4.9) and in separate sections for the respondents who answered yes, no, or do not know to the question “Did you acquire COVID-19?” (Multimedia Appendix 1, survey instrument items 6.1, 7.3, and 8.3), the instrument asked the question, “Did you have symptoms related to COVID-19? (answer options: Yes/No),” offering a checklist of 20 symptoms (new or increased cough, fever >38.0 °C, new or increased shortness of breath, sore throat, stuffy nose, runny nose, chest pain, sneezing, wheezing, headache, muscle aches, loss of taste, loss of smell, conjunctivitis or pink eye, confusion, seizures, weakness, and other).

For 65 RD-related symptoms grouped into 18 categories, the instrument asked the questions “Please check if you had any of the following symptoms before the COVID-19 pandemic began in the USA. Think about your symptoms in January-February 2020. Check all that apply” (answer options: yes or no) and “Did anything change after the beginning of the pandemic (March 2020)?” (If the previous answer was “No,” answer options: yes or no; if the previous answer was “Yes,” answer options: symptom absent, less severe, same severity, or more severe; Multimedia Appendix 1, survey instrument item 4.6).

For 91 medications and treatments grouped into 15 categories, the instrument asked the questions “Please check if you used any of the treatments before the COVID-19 pandemic began in the USA. Think about the medications you took or treatments you routinely received in January-February 2020. Check all that apply” (answer options: yes or no) and “Did anything change after the beginning of the pandemic (March 2020)?” (If the previous answer was “No,” answer options: yes or no; if the previous answer was “Yes,” answer options: treatment not used, lower dosage, same dosage, or higher dosage; Multimedia Appendix 1, survey instrument item 4.7).

We computed odds ratios (ORs) and their 95% CIs to illustrate the strength of the association between self-reported infection and specific COVID-19–related symptoms as well as the number of symptoms.

To assess changes in the prevalence or intensity of a specific COVID-19 symptom, we compared the number of patients who reported the symptom appearing during the pandemic (ie, the symptom was absent before the beginning of the pandemic but was present afterward) with the number of patients who reported the symptom disappearing (ie, the symptom was present before the beginning of the pandemic but was not present afterward) and used an exact binomial test of the null hypothesis that the 2 counts were equal (ie, the parameter of the binomial distribution was 0.5).

To assess changes in the prevalence and intensity of a specific RD symptom, we compared the number of patients who reported the symptom appearing or increasing in intensity during the pandemic with the number of patients who reported the symptom disappearing or decreasing in intensity and used the exact binomial test of the null hypothesis that the 2 counts were equal. We used the same logic and statistical test to assess the changes in the prevalence of the use or dosage of a specific medication. We used heat maps to summarize the changes in COVID-19–associated symptoms, RD symptoms, and RD treatment before and after the beginning of the pandemic.

The instrument asked the question, “Do you have other diseases or complications related to the rare disease? Check all that apply,” offering a list of 26 comorbidity categories (Multimedia Appendix 1, survey instrument item 4.8).

We computed ORs and their 95% CIs to illustrate the strength of the association between self-reported infection and specific comorbidities, reporting the exact *P* value for the test of the null hypothesis of no association (ie, OR=1). We regarded a finding as statistically significant if the statistical testing of a specified null hypothesis yielded a *P* value <.05.

## Results

Enrollment of participants for this study began on May 2, 2020, and ended on December 15, 2020; a total of 3413 respondents completed the survey. Most of the surveys (2643/3413, 77.44%) were completed between May and July (Figure S1 in Multimedia Appendix 1). Respondents approximately represented the geographic distribution of the US population (Table S1 in Multimedia Appendix 1): 25.76% (870/3377) were from the Midwest (21% expected) [69], 18.86% (637/3377) from the Northeast (18% expected), 33.43% (1129/3377) from the South (38% expected), and 21.94% (741/3377) from the West (24% expected). The diagnoses studied by the RDCRN accounted for 61.27% (2091/3413); and the most common diagnoses were myasthenia gravis (594/2091, 28.41%), amyotrophic lateral sclerosis (289/2091, 13.82%), eosinophilic esophagitis or eosinophilic gastrointestinal disease below the esophagus (203/2091, 9.71%), mitochondrial disease (174/2091, 8.32%), cystic fibrosis (87/2091, 4.16%), and primary ciliary dyskinesia (67/2091, 3.2%).

Two-thirds (2212/3413, 64.81%) of the respondents were female. Only 1.93% (66/3413) of the respondents reported being exclusively Black, 6.36% (217/3413) of the respondents reported other races or >1 race, and 89.01% (3038/3413) of the respondents were White (Table S2 in Multimedia Appendix 1). Approximately 5% (156/3413, 4.57%) reported Hispanic or Latino ethnicity, whereas most (2844/3413, 83.33%) of the respondents did not report ethnicity. Most of the respondents (2646/3413, 77.53%) were adults aged ≥25 years (Table S2 in Multimedia Appendix 1). The distribution by sex, race and ethnicity, and age was similar across disease categories, with the exception that most male respondents with eosinophilic esophageal or gastrointestinal diseases were aged <25 years. More detailed information on the demographic characteristics of the respondents by RD category is provided in Table S3 in Multimedia Appendix 1.

Overall, 80.6% (2751/3413) of the respondents reported that they had not acquired the COVID-19 infection, 2.08% (71/3413) of the respondents reported having acquired it, and 16.58% (566/3413) of the respondents did not know. Of the 71 respondents who reported acquiring the infection, 51 (72%) reported having a positive laboratory test. By contrast, only 15.2% (86/566) of the respondents who did not know reported having been tested (no positive test). We estimated that the self-reported cases represent the incidence over a total of 3219 person-years at risk, with an average annual incidence rate of 2.2% (95% CI 1.7%-2.8%).

**Figure 1:**
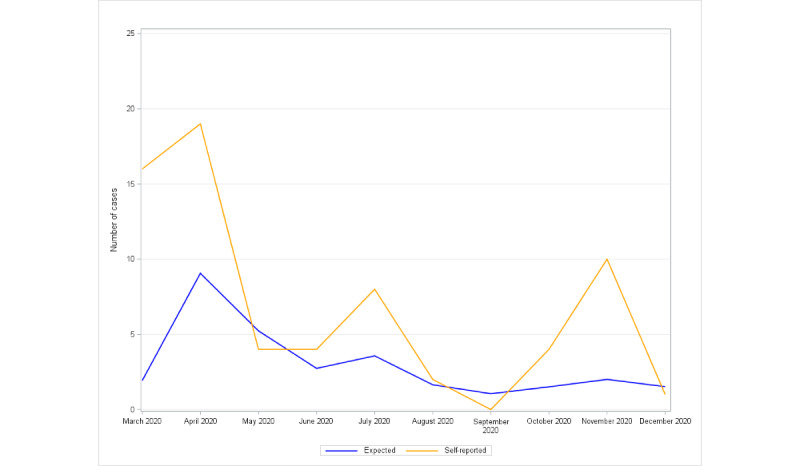
National survey of the impact of the COVID-19 pandemic on people with rare diseases (May 2, 2020, to December 15, 2020; N=3413). Number of self-reported COVID-19 cases by month, compared with the numbers expected on the basis of the monthly incidence rates reported by the New York Times for the entire US population.

Self-reported COVID-19 cases were twice as many as would be expected on the basis of the population incidence rates (71 vs 30.3; *P<*.001) and were concentrated in March to April 2020 (37 vs 11 expected) and in October to December 2020 (15 vs 5 expected; Figure 1), whereas self-reported cases were only slightly higher than expected in May to September 2020 (19 vs 14.2).

**Figure 2:**
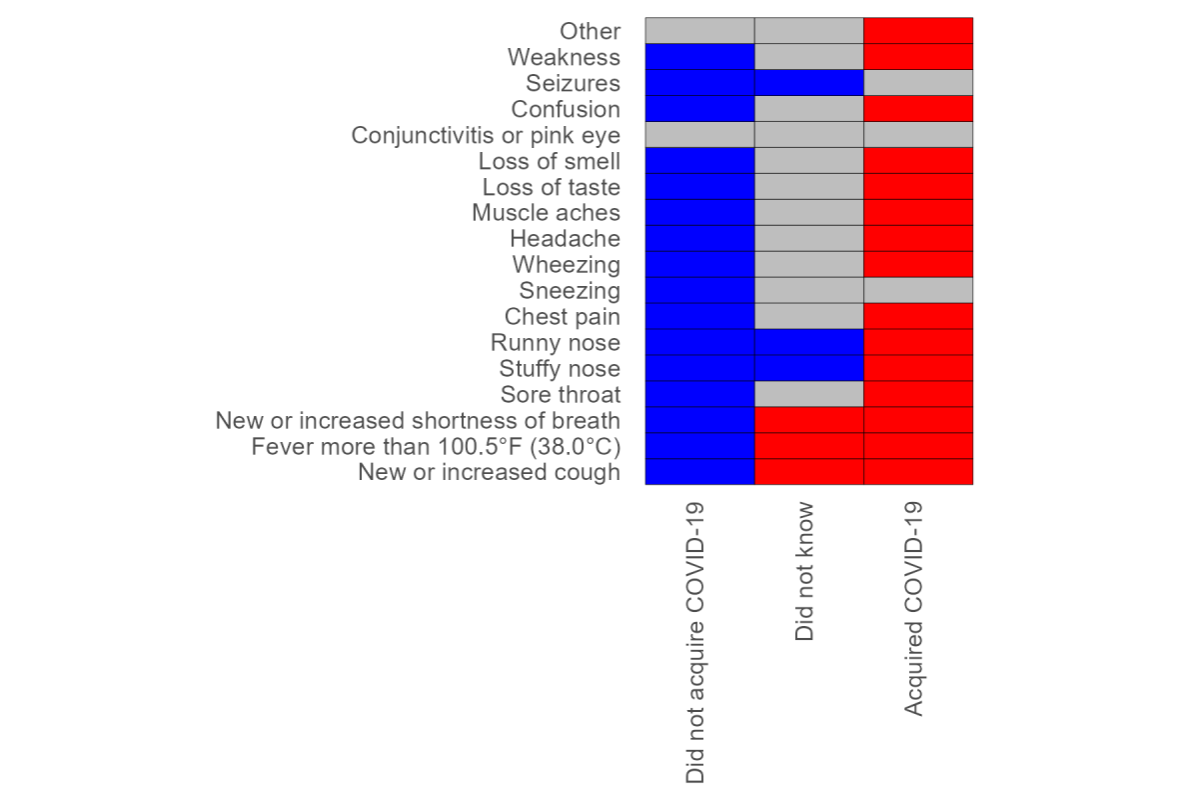
National survey of the impact of the COVID-19 pandemic on people with rare diseases (RDs; May 2, 2020, to December 15, 2020; N=3413). Heat map of pandemic-associated changes in the prevalence of COVID-19 symptoms. Statistical significance of item-specific changes based on an exact binomial test of the null hypothesis that positive changes (ie, symptom appearing or increasing in intensity and medication starting or dosage increasing during the pandemic) were equal in number to negative changes (ie, symptom disappearing or decreasing in intensity and medication discontinued or dosage decreasing during the pandemic). Respondents are categorized according to their answer to the question “Did you acquire COVID-19?”. Blue boxes indicate statistically significant decrease; grey boxes indicate statistically non-significant change; red boxes indicate statistically significant increase.

Approximately all respondents who acquired COVID-19 had symptoms (66/71, 93%). For 15 (83%) of the 18 symptoms, they reported experiencing the symptom more often after the beginning of the pandemic than before. Respondents who reported not acquiring COVID-19 had symptoms less often after the beginning of the pandemic than before for 16 (89%) of the 18 symptoms. The respondents who did not know displayed an intermediate pattern (Figure 2).

Specific COVID-19–related symptoms were strongly associated with self-reported infection; loss of taste (OR 38.9, 95% CI 22.4-67.6) or smell (OR 30.6, 95% CI 17.7-53.1), high fever (OR 14.5, 95% CI 8.7-24.1), and confusion (OR 12.7, 95% CI 6.80-23.7) were the strongest correlates of self-reported infection (Table 1).

Reporting multiple COVID-19–related symptoms was strongly associated with acquiring COVID-19 (>9 symptoms vs none: OR 82.5, 95% CI 29-234 and 5-9 symptoms: OR 44.8, 95% CI 18.7-107). Those who did not know were also more likely to report COVID-19–related symptoms and multiple symptoms than those who reported not acquiring the infection (Table 1). Respondents who acquired COVID-19 had demographic characteristics like others; most of the respondents were female (53/71, 75%), non-Hispanic White (59/71, 83%), and representative of the distribution of RD diagnoses reported by all respondents (results not shown in detail). The survey investigated 36 possible RD comorbidities, and 58.54% (1998/3413) of the respondents reported ≥1 comorbidities. Those who acquired COVID-19 (OR 1.60, 95% CI 1.00-2.50) and those who did not know (OR 1.40, 95% CI 1.20-1.70) were more likely to report RD comorbidities than those who did not acquire the infection. Individual associations between RD comorbidities and self-reported COVID-19 infection were weak (Table S4 in Multimedia Appendix 1).

**Figure 3:**
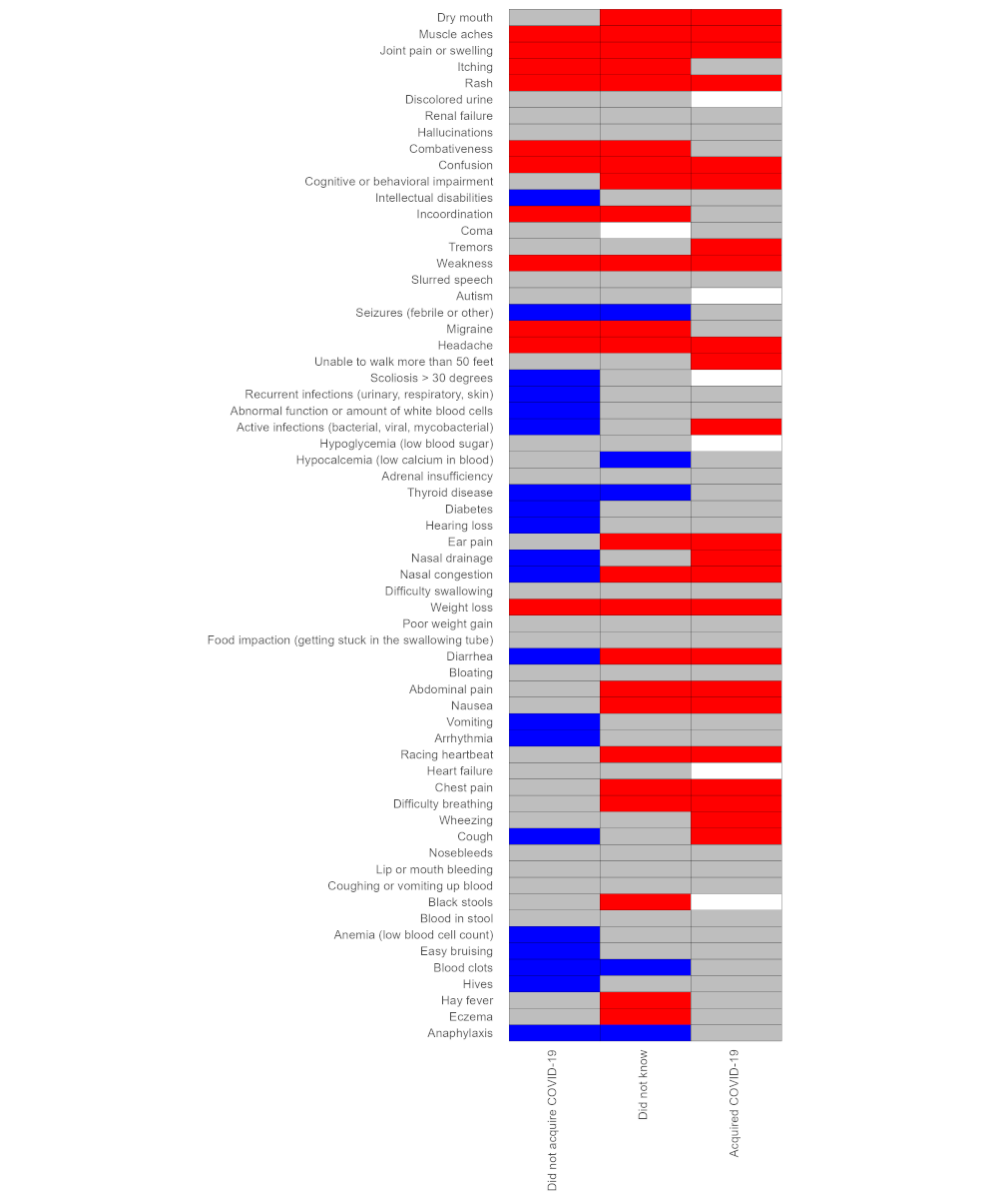
National survey of the impact of the COVID-19 pandemic on people with rare diseases (May 2, 2020, to December 15, 2020, N=3413). Heatmap of pandemic-associated changes in the prevalence and intensity of RD-associated symptoms. Statistical significance of symptom-specific changes based on an exact binomial test of the null hypothesis that positive changes (ie, symptom appearing or increasing in intensity during the pandemic) were equal in number to negative changes (ie, symptom disappearing or decreasing in intensity during the pandemic). Respondents are categorized according to their answer to the question “Did you acquire COVID-19?”. Blue boxes indicate statistically significant decrease; grey boxes indicate statistically non-significant change; red boxes indicate statistically significant increase; white boxes indicate inadequate data.

Respondents who acquired the infection reported a significantly increased occurrence or severity for 35% (23/65) of the RD-related symptoms from before the beginning of the COVID-19 pandemic to survey completion, no change for 52% (34/65) of the symptoms, and no significant decrease for any symptoms. Those who did not know reported significantly increased occurrence or severity for 37% (24/65) of the symptoms, no change for 51% (33/65) of the symptoms, and a significant decrease for 8% (5/65) of the symptoms. Those who did not acquire COVID-19 had increased occurrence or severity for 17% (11/65) of the symptoms, no change in 49% (32/65) of the symptoms, and significant decreases for 31% (20/65) of the symptoms (Figure 3).

**Figure 4:**
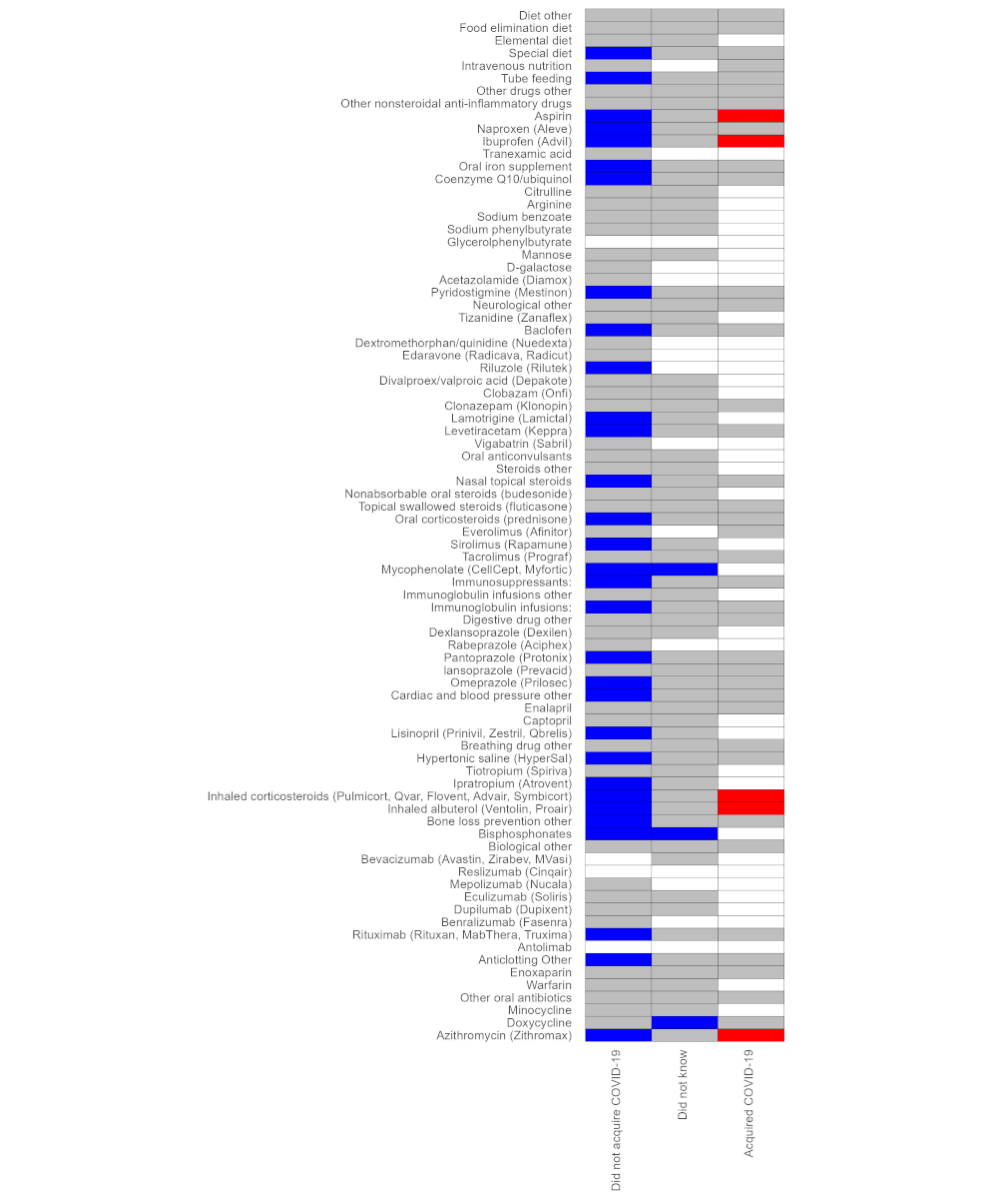
National survey of the impact of the COVID-19 pandemic on people with rare diseases (May 2, 2020, to December 15, 2020, N=3413). Heatmap of pandemic-associated changes in use and dosage of medications. Statistical significance of item-specific changes based on an exact binomial test of the null hypothesis that positive changes (ie, medication starting or dosage increasing during the pandemic) were equal in number to negative changes (ie, medication discontinued or dosage decreasing during the pandemic). Respondents are categorized according to their answer to the question “Did you acquire COVID-19?”. Blue boxes indicate statistically significant decrease; grey boxes indicate statistically non-significant change; red boxes indicate statistically significant increase; white boxes indicate inadequate data.

Respondents who acquired the infection reported significantly increased frequency or dosage for 5% (5/91) of the medications (azithromycin, inhaled albuterol, inhaled glucocorticoids, ibuprofen, and aspirin), whereas the frequency or dosage of other medications did not change significantly. Those who did not know did not have significant changes for most medications but had a significant decrease in frequency or dose for 3% (3/91) of the medications. Those who did not acquire COVID-19 reported no significant change for 51% (46/91) of the medications and significant decreases in use or dosage for 34% (31/91) of the medications (Figure 4).

Among those who reported acquiring the infection, the COVID-19 illness experience was not severe; symptoms lasting on average <30 days; hospitalization (7/71, 10%), and mechanical ventilator support (4/71, 6%) were relatively uncommon. However, 1 respondent in this group died, and many reported that either their RD complicated the COVID-19 illness (30/71, 44%) or that the infection worsened their baseline symptoms (39/71, 55%; Table 2).

The pandemic affected access to health care: 61% (42/69) of the respondents who acquired COVID-19 and 75.02% (2382/3175) of the other respondents experienced delays in obtaining an appointment, their appointment was done using telemedicine, or their appointment was postponed (Table 3).

In addition, access to treatment for the RD was affected. On the basis of nonmissing values, 25.99% (715/2751) of the respondents who did not acquire COVID-19, 32.7% (185/566) of the respondents who did not know, and 41% (29/71) of the respondents who acquired COVID-19 experienced delays in obtaining treatment or their therapies were interrupted during the pandemic. Access to food was uninterrupted for most of the respondents, but as many as 26% (18/70) of those who acquired COVID-19 had delays or an interruption in the supply of specialized diets (Table 3). The most pronounced disruption was in access to specialized treatment such as physical or speech therapy, with approximately 56% (32/57) of the respondents experiencing an interruption of treatment. Respondents who acquired COVID-19 reported more frequently (11/68, 16%) experiencing health events that would ordinarily require hospitalization but were managed without admission. Moreover, about 1 (13/67, 19%) of out 5 respondents reported that stay-at-home orders affected their mood or behavior in a way that required medical attention, and most of the respondents (480/2717, 17.67% to 28/71, 39%) noted that they or immediate family members sought professional support to cope with stress or anxiety, with the largest proportion (28/71, 39%) among those who acquired COVID-19 (Table 3). For all but 2 items in Table 3, the variation in responses across the 3 groups defined by self-reported COVID-19 status was statistically significant. Six deaths were reported, but 5 were among the respondents who did not acquire COVID-19.

**Table 1:** National survey of the impact of COVID-19 on people with rare diseases (May 2, 2020, to December 15, 2020; N=3413). COVID-19–related symptoms reported by survey respondents and association with answers to the question “Did you acquire COVID-19?” Those who responded “No” are the reference group.

		Acquired COVID-19 (n=71)		Did not know (n=566)			Did not acquire COVID-19 (n=2751), n (%)
		Values, n (%)	OR^a^ (95% CI)	Values, n (%)	OR (95% CI)		
**Specific symptom**							
	New or increased cough	37 (52)	10.7 (6.60-17.4)	201 (35.5)	5.40 (4.40-6.70)	253 (9.2)	
	Fever > 38.0 °C	29 (41)	14.5 (8.70-24.1)	145 (25.6)	7.20 (5.60-9.40)	125 (4.54)	
	New or increased shortness of breath	33 (46)	12.0 (7.30-19.5)	151 (26.7)	5.00 (4.00-6.40)	186 (6.76)	
	Sore throat	28 (39)	6.20 (3.80-10.2)	190 (33.6)	4.80 (3.90-6.00)	260 (9.45)	
	Stuffy nose	23 (32)	2.90 (1.80-4.90)	157 (27.7)	2.30 (1.90-2.90)	388 (14.10)	
	Runny nose	18 (25)	2.20 (1.30-3.80)	155 (27.4)	2.40 (2.00-3.00)	367 (13.34)	
	Chest pain	26 (37)	11.8 (7.10-19.8)	89 (15.7)	3.80 (2.90-5.10)	128 (4.65)	
	Sneezing	6 (8)	1.00 (0.40-2.40)	97 (17.1)	2.30 (1.80-3.00)	225 (8.18)	
	Wheezing	15 (21)	4.10 (2.30-7.50)	98 (17.3)	3.20 (2.50-4.20)	167 (6.07)	
	Headache	46 (65)	10.1 (6.10-16.6)	222 (39.2)	3.50 (2.90-4.30)	425 (15.45)	
	Muscle aches	41 (58)	8.00 (5.00-13.0)	196 (34.6)	3.10 (2.50-3.80)	400 (14.54)	
	Loss of taste	29 (41)	38.9 (22.4-67.6)	50 (8.8)	5.50 (3.60-8.20)	48 (1.74)	
	Loss of smell	27 (38)	30.6 (17.7-53.1)	50 (8.8)	4.80 (3.30-7.20)	54 (1.96)	
	Conjunctivitis or pink eye	5 (7)	7.10 (2.70-18.9)	35 (6.2)	6.20 (3.70-10.2)	29 (1.05)	
	Confusion	15 (21)	12.7 (6.80-23.7)	45 (8.0)	4.10 (2.70-6.10)	57 (2.07)	
	Seizures	1 (1)	0.90 (0.10-6.30)	8 (1.4)	0.90 (0.40-1.80)	45 (1.64)	
	Weakness	33 (46)	6.30 (3.90-10.2)	152 (26.9)	2.70 (2.10-3.30)	333 (12.1)	
	Other	20 (28)	10.6 (6.10-18.5)	50 (8.8)	2.60 (1.80-3.70)	98 (3.56)	
**Number of symptoms reported**							
	1-2	6 (8)	4.00 (1.30-12.5)	24 (4.2)	0.40 (0.20-0.60)	435 (15.81)	
	3-4	11 (15)	11.3 (4.10-30.8)	59 (10.4)	1.40 (1.00-1.90)	284 (10.32)	
	5-9	37 (52)	44.8 (18.7-107)	167 (29.5)	4.80 (3.80-6.00)	241 (8.76)	
	>9	11 (15)	82.4 (29.0-234)	61 (10.8)	10.7 (7.00-16.4)	39 (1.42)	

^a^OR: odds ratio.

**Table 2:** National survey of the impact of COVID-19 on people with rare diseases (May 2, 2020, to December 15, 2020). Selected characteristics of survey respondents who reported acquiring COVID-19 (n=71).

Characteristics	Values
Sex: female, n (%)	53 (75)
Age (y), mean (SD; range)	44 (21; 1-82)
Race and ethnicity: White, non-Hispanic or Latino, n (%)	59 (83)
Duration of symptoms (days), median (IQR)	16 (9-30)
Symptoms persisting through the survey date, n (%)	24 (37)
Sought care at an emergency department or urgent care center, n (%)	28 (39)
Was admitted to a hospital, n (%)	7 (10)
Required supplemental oxygen, n (%)	12 (17)
Required intubation and mechanical ventilation, n (%)	4 (6)
Received investigational drugs or participated in a clinical trial, n (%)	9 (13)
Rare disease complicated the COVID-19 illness, n (%)	30 (44)
COVID-19 illness worsened the rare disease symptoms, n (%)	39 (55)

**Table 3:** National survey of the impact of COVID-19 on people with rare diseases (May 2, 2020, to December 15, 2020; N=3413). Responses to survey items addressing the impact of the pandemic on survey respondents and their families, according to the answer to the question “Did you acquire COVID-19?”

Survey item and responses		Acquired COVID-19 (n=71), n (%)	Did not know (n=566), n (%)	Did not acquire COVID-19 (n=2751), n (%)	*P* value^a^	
**Were you able to continue seeing your health care provider?**						.10
	Yes, without problems	27 (39.13)	130 (23.81)	663 (25.22)		
	Yes, but experienced delays in obtaining and appointment	3 (4.35)	32 (5.86)	157 (5.97)		
	Yes, but my appointment was done via telemedicine	25 (36.23)	241 (44.14)	1214 (46.18)		
	No, appointment was put on hold	14 (20.29)	143 (26.19)	595 (22.63)		
**Were you able to continue your treatment?**						<.001
	Yes, without problems	39 (57.35)	352 (65.55)	1886 (72.51)		
	Yes, but experienced delays in obtaining treatment	8 (11.76)	82 (15.27)	319 (12.26)		
	No, treatment was interrupted	21 (30.88)	103 (19.18)	396 (15.22)		
**Were you able to maintain your diet?**						<.001
	Yes, without problems	52 (74.29)	417 (76.51)	2231 (84.48)		
	Yes, but experienced delays in obtaining treatment	11 (15.71)	96 (17.61)	307 (11.62)		
	No, supply of needed food was interrupted and my diet suffered from it	7 (10)	32 (5.87)	103 (3.9)		
**Were you able to continue specialized treatment?**						.07
	Yes, without problems	31 (54.39)	250 (56.56)	1082 (51.3)		
	Yes, but experienced delays in obtaining treatment	1 (1.75)	26 (5.88)	179 (8.49)		
	No, treatment was interrupted	25 (43.86)	166 (37.56)	848 (40.21)		
**Did you experience a medical event for which you would ordinarily be hospitalized, but because of COVID-19 you were managed without hospitalization?**						<.001
	No	52 (76.47)	468 (84.02)	2476 (92.84)		
	Yes	11 (16.18)	56 (10.05)	154 (5.77)		
	Unknown	5 (7.35)	33 (5.92)	37 (1.39)		
**Have stay-at-home orders in your area affected your mood or behavior in a way that requires medical attention?**						<.001
	No	48 (71.64)	414 (73.53)	2193 (81.92)		
	Yes	13 (19.4)	122 (21.67)	399 (14.9)		
	Unknown	6 (8.96)	27 (4.8)	85 (3.18)		
**Have you or members of your family sought professional support coping with stress or anxiety because of the COVID-19 pandemic?**						<.001
	No	43 (60.56)	422 (74.82)	2237 (82.33)		
	Yes	28 (39.44)	142 (25.18)	480 (17.67)		

^a^Test of the null hypothesis that proportions do not vary across the 3 groups. Percentages are reported based on nonmissing values. Missing values are not reported for readability.

## Discussion

### Principal Findings

This study reports results from the largest survey assessing the impact of the COVID-19 pandemic on people living with RDs; >3000 individuals representing a wide range of RDs participated in the study. Women, adults, and White non-Hispanic individuals were overrepresented. Recruitment and data collection procedures may have discouraged the participation of minority groups, although the causes of their underrepresentation are probably broader. For instance, the racial and ethnic profile of the respondents living with myasthenia gravis was similar to that of the participants of a national registry [31], but it is possible that the registry also underrepresented minority groups. Similarly, in the National Amyotrophic Lateral Sclerosis Registry, of the estimated 25,000 prevalent cases in 2017, 64.97% (16,127/24,821) were White, 5.92% (1469/24,821) were Black. [70]. In 2012 pediatric Medicaid records, 59.38% (2872/4836) of the patients with eosinophilic esophagitis were White and 15.47% (748/4836) were Black, whereas race was missing for 11.81% (571/4836) of the patients [71]. Our survey was widely advertised and relied on outreach by patient advocacy groups. Participation of >1300 respondents with an RD not studied by the RDCRN suggests that our outreach was effective in the RD community.

Underrepresentation of minority groups may be related to the globally recognized barriers to access to RD diagnosis, specialized care and social support [72,73], and other determinants of lack of participation in research [74,75]. Spurred by these findings, the RDCRN has established a cross-consortia Diversity Committee to identify actions to support and expand the participation and engagement of research participants, family members, advocates, and research staff from underrepresented or marginalized communities.

COVID-19 infection was reported by a few respondents (71/3413, 2.08%), but the number was larger than expected. The excess cases were concentrated in the first months of the pandemic and at the end of the data collection period. Thus, individuals who experienced COVID-19 early may have been more motivated to participate in the study. Likewise, survey advertisements in late 2020 could have motivated the participation of patients with an RD who were experiencing the next surge in the epidemic in the United States. Although selection is possible, the number of self-reported infections must underestimate the incidence of COVID-19 in the RD community, as underscored by the large proportion (566/3413, 16.58%) of those who did not know if they acquired the infection. This group reported COVID-19–related symptoms far more frequently than those who did not acquire COVID-19. Given the lack of access to testing early during the pandemic, many individuals with mild COVID-19 infection may have never been diagnosed. An NIH serosurvey of a sample of the US population estimated that 5 undiagnosed SARS-CoV-2 infections occurred for every COVID-19 case, and that approximately 17 million undiagnosed infections occurred in the United States by mid-July 2020 [76]. These findings have been confirmed in independent assessments [77,78]. Thus, despite the uncertainty about the true number of cases, our results indicate that COVID-19 has had a large impact on the RD community.

Self-reported COVID-19 was not associated with specific RD conditions, but RD comorbidities were associated with increased odds of acquiring COVID-19. Respondents who acquired the infection described a mild disease, with few requiring hospitalization or critical care and only 1 death reported. A mild course of COVID-19 infections has been reported among patients with hereditary hemorrhagic telangiectasia in Italy [50], rare endocrine diseases in a European registry study [38], lysosomal storage diseases in London [79], and Gaucher disease in New York [42]; in a large group of patients with RD in Brazil [43]; and among people with myasthenia gravis in a subanalysis of this survey [52]. These reassuring findings are in contrast with higher COVID-19–related mortality among patients with RD in Hong Kong hospitals compared with in-patients from the general population [37] and people with neurological and neurodevelopmental diseases in a Genomics England cohort study [33]. We observed that the frequency and severity of RD-related symptoms worsened during the pandemic among those who reported acquiring COVID-19 or did not know compared with those who were uninfected, indicating that COVID-19 complicated the underlying RD.

We found a greater use of selected medications among those who acquired COVID-19, little change among those who did not know, and decrease in use and dosage of many medications among those who did not acquire COVID-19. We did not expect the uninfected respondents to report reduced medication use. The need for some medications may have normally decreased between the winter before the beginning of the pandemic and the early summer when most surveys were completed. Seasonal variation exists in the use of antibiotics and prescription drugs for asthma, cystic fibrosis, and other respiratory illnesses [80-83] but not for other medications evaluated in the survey. It is possible that the pandemic decreased access to care and reduced medication use among those who were not in immediate need, whereas respondents with confirmed or possible COVID-19 infection continued to use their medications. This interpretation is not fully supported by the survey responses, which suggest that the pandemic interfered with access to regular health care, treatment for the RD, and hospitalization less frequently with respondents who did not acquire COVID-19. Several studies involving patients with RD have reported reduced access to care [36,40,43,45,52], and of patient organizations [84-88] and providers [79,89-93] have reported increased difficulties in delivering care and services, negatively affecting patients with RD. However, some reports indicate that the rise in telehealth services has had a positive impact in the RD community [43,47,90]. Access to prescription medications for asthma and chronic obstructive pulmonary disease in Great Britain initially briefly increased and then declined to below prepandemic levels during 2020 [94].

We found that the pandemic caused greater mood changes, anxiety, and stress in both respondents and their family members to an extent that required medical attention. These effects were experienced by a significant proportion of all respondents.

### Strengths and Limitations

This study had certain limitations. First, we could not determine whether respondents were representative of the RD population. However, this concern is mitigated by the representative geographic distribution and the large number of diagnoses reported by the respondents: 48.17% (1644/3413) reported a diagnosis that was not studied by the RDCRN, suggesting that the outreach effort was effective in the RD community at large. Demographics and self-reported outcomes did not vary considerably across RDs, suggesting that the same selection forces affected all respondents.

Second, the information collected pertains mostly to adult, White non-Hispanic people with RDs. Thus, we could not examine racial or ethnic disparities in the impact of the COVID-19 pandemic.

Third, the main motivation for conducting this survey was that most studies examining the impact of COVID-19 would focus on the general population and not on people living with an RD. We did not have access to a suitable comparison group; therefore, this report is based only on internal comparisons. Finally, data collection was based on self-reports, and we did not have external information to validate our observations. Our inference is based on internal comparisons, the validity of which relies on the assumption that between-group differences are unbiased.

Despite these limitations, this survey had substantial strengths. This is the largest survey conducted to learn about the experience of people living with RDs during the COVID-19 pandemic. The large number of respondents allowed a precise assessment of the prevalence and changes in symptoms and medications used during the early phase of the pandemic and robust internal comparisons among respondents who reported acquiring COVID-19 or were uncertain but had symptoms (ie, may have had an infection without a clinical or laboratory diagnosis). The survey provided a necessary assessment of the impact of the pandemic on the families of people living with RDs. Most participants stated their willingness to participate in follow-up surveys and future research. Thus, this survey provides a basis for evaluating the longitudinal impact of the pandemic on respondents and their families.

### Conclusions

In conclusion, the incidence of self-reported COVID-19 infection in this survey of people living with RDs was higher than expected based on population rates, and many respondents were unsure if they had acquired the infection. COVID-19–related symptoms were strongly associated with self-reported infection and with unknown infection status. Although the clinical severity of the infection was not high, self-reported COVID-19 was moderately associated with RD comorbidities and was strongly associated with increased prevalence and severity of RD-related symptoms as well as greater use and dosage of certain medications. The pandemic negatively affected access to health care, RD treatment, and hospitalization; it caused mood changes and greater anxiety for respondents and their family members, requiring medical attention for some. These effects were experienced not only by those who acquired the infection but also by those who did not acquire COVID-19 during the early months of the pandemic or were unsure about it. Continued surveillance of this population is needed to inform interventions to mitigate the impact of COVID-19 and better prepare people with RDs, the health care system, and society for a future pandemic.

## Appendix

Multimedia Appendix 1 Supplemental materials and survey instrument.

## Abbreviations

IRB: institutional review board
NIH: National Institutes of Health
OR: odds ratio
RD: rare disease
RDCRN: Rare Diseases Clinical Research Network
REDCap: Research Electronic Data Capture
WHO: World Health Organization

## Data Availability

All participant-level data elements necessary to reproduce the analyses presented in the manuscript will be made available upon request. No direct personal identifiers of survey participants will be made available. Survey responses and computed variables used for the analyses presented in the manuscript will be made available in Excel workbooks (Microsoft Corporation) and SAS data sets (SAS Institute). Prospective users will be required to file a data access application including the objectives of data access and the intended use (including a data analysis plan). A data access committee will review the application and determine whether to grant access to the data. Users may be required to sign a data use agreement before obtaining access to the data. Data and supporting documents will be available through the Rare Diseases Clinical Research Network Data Repository (under development) as soon as the Data Repository is active. Please check the Rare Diseases Clinical Research Network website for updates.
