# Supplementary material for: Impact of the COVID-19 Pandemic on People Living With Rare Diseases and Their Families: Results of a National Survey
Source: JMIR Public Health Surveill. 2024 Feb 14;10:e48430. doi: 10.2196/48430 (PMC10868638; doi:10.2196/48430)
Supplement: Multimedia Appendix 1 [file publichealth_v10i1e48430_app1.docx]

Supplement

Figure S1. Pareto chart displaying the numbers of surveys completed by month, with a line indicating the cumulative number.

Table S1. Distribution of completed surveys by state compared with U.S. population percent.

Table S2. Demographic characteristics of the respondents.

Table S3. Demographic characteristics of survey respondents in specific diagnostic groups.

Table S4. Select Rare Disease-Associated Conditions and Self-Reported COVID-19 Status.

RDCRN COVID-19 Survey Instrument.


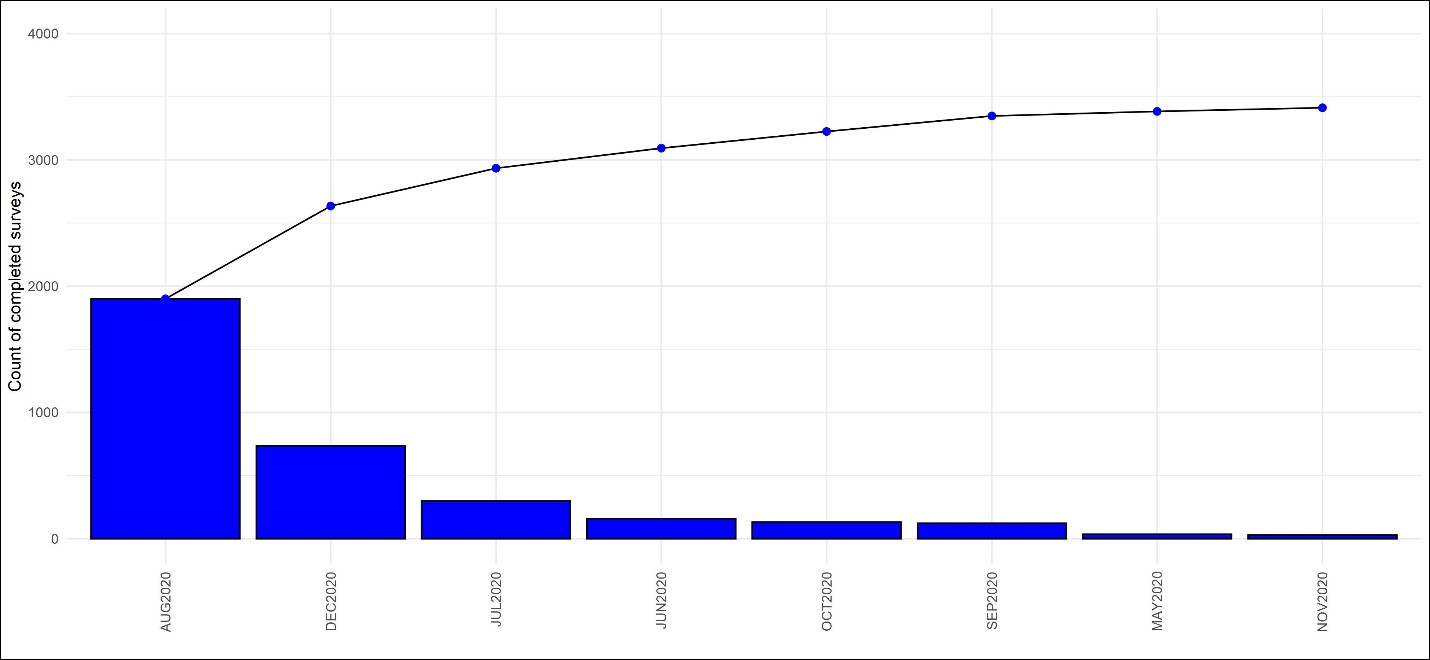


National survey of the impact of COVID-19 on people with rare diseases (05/02/2020-12/15/2020, n = 3413). Monthly and cumulative distribution of participants.

| Table S1. National survey of the impact of COVID-19 on people with rare diseases (05/02/2020-12/15/2020, n = 3413). Distribution of survey respondents and US population by state of residence. | | | | | | | |
| --- | --- | --- | --- | --- | --- | --- | --- |
| State | Surveys completed | Percent of surveys | U.S. Population percent | State | Surveys completed | Percent of surveys | U.S. Population percent |
| California | 296 | 8.8% | 11.8% | Oregon | 66 | 2.0% | 1.3% |
| Texas | 202 | 6.0% | 8.9% | Oklahoma | 28 | 0.8% | 1.2% |
| Florida | 207 | 6.1% | 6.6% | Connecticut | 53 | 1.6% | 1.1% |
| New York | 170 | 5.0% | 6.0% | Utah | 26 | 0.8% | 1.0% |
| Pennsylvania | 143 | 4.2% | 3.9% | Iowa | 53 | 1.6% | 1.0% |
| Illinois | 151 | 4.5% | 3.8% | Nevada | 24 | 0.7% | 0.9% |
| Ohio | 147 | 4.3% | 3.5% | Arkansas | 14 | 0.4% | 0.9% |
| Georgia | 104 | 3.1% | 3.3% | Mississippi | 12 | 0.4% | 0.9% |
| North Carolina | 101 | 3.0% | 3.2% | Kansas | 38 | 1.1% | 0.9% |
| Michigan | 115 | 3.4% | 3.0% | New Mexico | 12 | 0.4% | 0.6% |
| New Jersey | 92 | 2.7% | 2.8% | Nebraska | 15 | 0.4% | 0.6% |
| Virginia | 114 | 3.4% | 2.6% | Idaho | 19 | 0.6% | 0.6% |
| Washington | 108 | 3.2% | 2.3% | West Virginia | 25 | 0.7% | 0.5% |
| Arizona | 78 | 2.3% | 2.2% | Hawaii | 5 | 0.1% | 0.4% |
| Massachusetts | 87 | 2.6% | 2.1% | New Hampshire | 34 | 1.0% | 0.4% |
| Tennessee | 56 | 1.7% | 2.1% | Maine | 18 | 0.5% | 0.4% |
| Indiana | 86 | 2.5% | 2.1% | Montana | 13 | 0.4% | 0.3% |
| Missouri | 65 | 1.9% | 1.9% | Rhode Island | 11 | 0.3% | 0.3% |
| Maryland | 117 | 3.5% | 1.9% | Delaware | 14 | 0.4% | 0.3% |
| Wisconsin | 65 | 1.9% | 1.8% | South Dakota | 41 | 1.2% | 0.3% |
| Colorado | 76 | 2.2% | 1.8% | North Dakota | 7 | 0.2% | 0.2% |
| Minnesota | 87 | 2.6% | 1.7% | Alaska | 7 | 0.2% | 0.2% |
| South Carolina | 38 | 1.1% | 1.6% | District of Columbia | 11 | 0.3% | 0.2% |
| Alabama | 33 | 1.0% | 1.5% | Vermont | 15 | 0.4% | 0.2% |
| Louisiana | 29 | 0.9% | 1.4% | Wyoming | 11 | 0.3% | 0.2% |
| Kentucky | 38 | 1.1% | 1.4% | Puerto Rico | 4 | 0.1% | . |

| Table S2. National survey of the impact of COVID-19 on people with rare diseases (05/02/2020-12/15/2020, n = 3413). Demographic characteristics of survey respondents. | | |
| --- | --- | --- |
| Characteristic | Number of respondents | Percent % |
| Sex | | |
| Male | 1126 | 33.5% |
| Female | 2212 | 65.9% |
| Other | 12 | 0.4% |
| Choose not to answer | 7 | 0.2% |
| Race | | |
| American Indian or Native Alaskan only | 10 | 0.3% |
| Asian only | 65 | 2.0% |
| Black only | 66 | 2.0% |
| Native Hawaiian or Pacific Islander only | 2 | 0.1% |
| White only | 3038 | 91.5% |
| More than one race | 88 | 2.6% |
| Other | 52 | 1.6% |
| Ethnicity | | |
| Hispanic/Latino | 156 | 4.8% |
| Non-Hispanic or Latino | 2844 | 87.8% |
| Unknown | 87 | 2.7% |
| Choose not to answer | 152 | 4.7% |
| Age category | | |
| 0-14 years old | 460 | 13.6% |
| 15-24 years old | 286 | 8.4% |
| 25 years old | 2646 | 78.0% |

| Table S3. National survey of the impact of COVID-19 on people with rare diseases (05/02/2020-12/15/2020, n = 3413). Demographic characteristics of survey respondents in specific diagnostic groups (counts <10 are not reported). | | | |
| --- | --- | --- | --- |
| **Rare disease category** | **Characteristic** | **Number of respondents** | **Percent %** |
| Vasculitis | Sex: Female | 46 | 90% |
| Vasculitis | Race: White only | 43 | 86% |
| Vasculitis | Ethnicity: Non-Hispanic or Latino. | 44 | 94% |
| Vasculitis | Age category: 25+ years old | 49 | 96% |
| Rare lung diseases | Sex: Male | 11 | 12% |
| Rare lung diseases | Sex: Female | 84 | 88% |
| Rare lung diseases | Race: White only | 87 | 93% |
| Rare lung diseases | Ethnicity: Non-Hispanic or Latino. | 81 | 88% |
| Rare lung diseases | Age category: 25+ years old | 94 | 98% |
| Primary immune deficiencies | Sex: Male | 12 | 18% |
| Primary immune deficiencies | Sex: Female | 55 | 82% |
| Primary immune deficiencies | Race: White only | 61 | 91% |
| Primary immune deficiencies | Ethnicity: Non-Hispanic or Latino. | 55 | 92% |
| Primary immune deficiencies | Age category: 25+ years old | 57 | 85% |
| Mitochondrial diseases | Sex: Male | 48 | 28% |
| Mitochondrial diseases | Sex: Female | 120 | 71% |
| Mitochondrial diseases | Race: White only | 152 | 93% |
| Mitochondrial diseases | Ethnicity: Hispanic/Latino. | 10 | 6% |
| Mitochondrial diseases | Ethnicity: Non-Hispanic or Latino. | 139 | 85% |
| Mitochondrial diseases | Ethnicity: Choose not to answer. | 12 | 7% |
| Mitochondrial diseases | Age category: 0-14 years old | 34 | 20% |
| Mitochondrial diseases | Age category: 15-24 years old | 21 | 12% |
| Mitochondrial diseases | Age category: 25+ years old | 118 | 68% |
| Myasthenia Gravis | Sex: Male | 246 | 42% |
| Myasthenia Gravis | Sex: Female | 334 | 57% |
| Myasthenia Gravis | Race: Black only | 15 | 3% |
| Myasthenia Gravis | Race: White only | 528 | 93% |
| Myasthenia Gravis | Race: Other | 11 | 2% |
| Myasthenia Gravis | Ethnicity: Hispanic/Latino. | 19 | 3% |
| Myasthenia Gravis | Ethnicity: Non-Hispanic or Latino. | 489 | 88% |
| Myasthenia Gravis | Ethnicity: Unknown. | 17 | 3% |
| Myasthenia Gravis | Ethnicity: Choose not to answer. | 33 | 6% |
| Myasthenia Gravis | Age category: 25+ years old | 579 | 98% |
| Lysosomal disease | Sex: Male | 39 | 43% |
| Lysosomal disease | Sex: Female | 51 | 57% |
| Lysosomal disease | Race: White only | 82 | 89% |
| Lysosomal disease | Ethnicity: Non-Hispanic or Latino. | 81 | 90% |
| Lysosomal disease | Age category: 0-14 years old | 19 | 20% |
| Lysosomal disease | Age category: 15-24 years old | 15 | 16% |
| Lysosomal disease | Age category: 25+ years old | 59 | 63% |
| Genetic Disorders of Mucociliary Clearance | Sex: Male | 37 | 33% |
| Genetic Disorders of Mucociliary Clearance | Sex: Female | 76 | 67% |
| Genetic Disorders of Mucociliary Clearance | Race: White only | 105 | 95% |
| Genetic Disorders of Mucociliary Clearance | Ethnicity: Non-Hispanic or Latino. | 100 | 93% |
| Genetic Disorders of Mucociliary Clearance | Age category: 0-14 years old | 23 | 20% |
| Genetic Disorders of Mucociliary Clearance | Age category: 15-24 years old | 18 | 16% |
| Genetic Disorders of Mucociliary Clearance | Age category: 25+ years old | 73 | 64% |
| Cystic Fibrosis | Sex: Male | 25 | 29% |
| Cystic Fibrosis | Sex: Female | 62 | 71% |
| Cystic Fibrosis | Race: White only | 85 | 98% |
| Cystic Fibrosis | Ethnicity: Non-Hispanic or Latino. | 73 | 90% |
| Cystic Fibrosis | Age category: 0-14 years old | 12 | 14% |
| Cystic Fibrosis | Age category: 25+ years old | 68 | 78% |
| ALS | Sex: Male | 155 | 54% |
| ALS | Sex: Female | 127 | 45% |
| ALS | Race: White only | 269 | 96% |
| ALS | Ethnicity: Non-Hispanic or Latino. | 241 | 87% |
| ALS | Ethnicity: Unknown. | 13 | 5% |
| ALS | Ethnicity: Choose not to answer. | 14 | 5% |
| ALS | Age category: 25+ years old | 280 | 98% |
| Eosinophilic diseases | Sex: Male | 103 | 53% |
| Eosinophilic diseases | Sex: Female | 90 | 46% |
| Eosinophilic diseases | Race: White only | 178 | 90% |
| Eosinophilic diseases | Ethnicity: Hispanic/Latino. | 10 | 5% |
| Eosinophilic diseases | Ethnicity: Non-Hispanic or Latino. | 175 | 90% |
| Eosinophilic diseases | Age category: 0-14 years old | 85 | 42% |
| Eosinophilic diseases | Age category: 15-24 years old | 41 | 20% |
| Eosinophilic diseases | Age category: 25+ years old | 75 | 37% |
| Brittle Bone Disorders | Sex: Female | 19 | 86% |
| Brittle Bone Disorders | Race: White only | 21 | 95% |
| Brittle Bone Disorders | Ethnicity: Non-Hispanic or Latino. | 18 | 82% |
| Brittle Bone Disorders | Age category: 25+ years old | 18 | 82% |
| Brain Vascular Malformation | Sex: Female | 14 | 74% |
| Brain Vascular Malformation | Race: White only | 18 | 95% |
| Brain Vascular Malformation | Ethnicity: Non-Hispanic or Latino | 18 | 95% |
| Brain Vascular Malformation | Age category: 25+ years old | 16 | 84% |
| Congenital and Perinatal Infections | Sex: Female | 10 | 63% |
| Congenital and Perinatal Infections | Race: White only | 16 | 100% |
| Congenital and Perinatal Infections | Ethnicity: Non-Hispanic or Latino. | 12 | 75% |
| Congenital and Perinatal Infections | Age category: 0-14 years old | 11 | 69% |
| Dystonia | Sex: Female | 21 | 88% |
| Dystonia | Race: White only | 23 | 96% |
| Dystonia | Ethnicity: Non-Hispanic or Latino. | 20 | 83% |
| Dystonia | Age category: 25+ years old | 23 | 96% |
| Developmental Synaptopathies | Sex: Male | 13 | 33% |
| Developmental Synaptopathies | Sex: Female | 27 | 68% |
| Developmental Synaptopathies | Race: White only | 37 | 93% |
| Developmental Synaptopathies | Ethnicity: Non-Hispanic or Latino. | 34 | 87% |
| Developmental Synaptopathies | Age category: 0-14 years old | 12 | 30% |
| Developmental Synaptopathies | Age category: 15-24 years old | 12 | 30% |
| Developmental Synaptopathies | Age category: 25+ years old | 16 | 40% |
| Congenital Disorders of Glycosylation | Race: White only | 15 | 83% |
| Congenital Disorders of Glycosylation | Ethnicity: Non-Hispanic or Latino. | 16 | 89% |
| Leukodystrophy | Sex: Male | 13 | 42% |
| Leukodystrophy | Sex: Female | 18 | 58% |
| Leukodystrophy | Race: White only | 23 | 79% |
| Leukodystrophy | Ethnicity: Non-Hispanic or Latino. | 21 | 70% |
| Leukodystrophy | Age category: 25+ years old | 22 | 71% |
| Inherited Neuropathies | Sex: Female | 32 | 82% |
| Inherited Neuropathies | Race: White only | 37 | 95% |
| Inherited Neuropathies | Ethnicity: Non-Hispanic or Latino. | 33 | 89% |
| Inherited Neuropathies | Age category: 25+ years old | 39 | 100% |
| Porphyrias | Sex: Female | 41 | 85% |
| Porphyrias | Race: White only | 46 | 94% |
| Porphyrias | Ethnicity: Non-Hispanic or Latino. | 40 | 91% |
| Porphyrias | Age category: 25+ years old | 46 | 94% |
| Rare Rett Syndrome, MECP2 Duplications, and Rett-related Disorders | Sex: Female | 33 | 94% |
| Rare Rett Syndrome, MECP2 Duplications, and Rett-related Disorders | Race: White only | 30 | 86% |
| Rare Rett Syndrome, MECP2 Duplications, and Rett-related Disorders | Ethnicity: Non-Hispanic or Latino. | 28 | 78% |
| Rare Rett Syndrome, MECP2 Duplications, and Rett-related Disorders | Age category: 0-14 years old | 15 | 42% |
| Rare Rett Syndrome, MECP2 Duplications, and Rett-related Disorders | Age category: 25+ years old | 14 | 39% |
| Urea Cycle Disorders | Sex: Male | 12 | 36% |
| Urea Cycle Disorders | Sex: Female | 21 | 64% |
| Urea Cycle Disorders | Race: White only | 29 | 91% |
| Urea Cycle Disorders | Ethnicity: Non-Hispanic or Latino. | 28 | 88% |
| Urea Cycle Disorders | Age category: 0-14 years old | 12 | 36% |
| Urea Cycle Disorders | Age category: 25+ years old | 12 | 36% |
| Other rare diseases | Sex: Male | 365 | 28% |
| Other rare diseases | Sex: Female | 917 | 71% |
| Other rare diseases | Race: Asian only | 39 | 3% |
| Other rare diseases | Race: Black only | 26 | 2% |
| Other rare diseases | Race: White only | 1147 | 90% |
| Other rare diseases | Race: More than one race | 39 | 3% |
| Other rare diseases | Race: Other | 24 | 2% |
| Other rare diseases | Ethnicity: Hispanic/Latino. | 67 | 5% |
| Other rare diseases | Ethnicity: Non-Hispanic or Latino. | 1093 | 88% |
| Other rare diseases | Ethnicity: Unknown. | 35 | 3% |
| Other rare diseases | Ethnicity: Choose not to answer. | 51 | 4% |
| Other rare diseases | Age category: 0-14 years old | 210 | 16% |
| Other rare diseases | Age category: 15-24 years old | 120 | 9% |
| Other rare diseases | Age category: 25+ years old | 970 | 75% |

| Table S4. National survey of the impact of COVID-19 on people with rare diseases (05/02/2020-12/15/2020, n = 3413). RD-associated conditions reported by survey respondents and association (ORs and 95%CI) with answers to the question “Did you acquire COVID-19?” Those who responded “No” are the reference group. | | | | | | | | | |
| --- | --- | --- | --- | --- | --- | --- | --- | --- | --- |
|  | Acquired COVID (N=71) | | | | Did not know (N=566) | | | | Did not acquire COVID (N=2,751) |
| Condition | N | OR (95%CI) | P-value | N | | OR (95%CI) | *P*-value | N | |
| Any associated condition | 41 | 1.60 (1.00-2.50) | .07 | 316 | | 1.40 (1.20-1.70) | <.001 | 1271 | |
| Coronary artery disease | 2 | 1.20 (0.30-4.90) | .82 | 18 | | 1.30 (0.80-2.30) | .28 | 66 | |
| Congenital heart disease | 0 | 0.60 (0.00-2.60) | .98 | 17 | | 1.80 (1.00-3.20) | .04 | 46 | |
| Hypertension | 1 | 0.20 (0.00-1.10) | .07 | 52 | | 1.10 (0.80-1.50) | .52 | 230 | |
| Other heart diseases | 0 | 0.40 (0.00-1.70) | .98 | 28 | | 2.00 (1.30-3.10) | .003 | 70 | |
| Diabetes | 4 | 1.10 (0.40-2.90) | .91 | 27 | | 0.90 (0.60-1.40) | .58 | 147 | |
| Hypothyroidism | 7 | 1.50 (0.70-3.30) | .33 | 62 | | 1.70 (1.20-2.30) | <.001 | 189 | |
| Adrenal Insufficiency | 2 | 1.30 (0.30-5.40) | .72 | 11 | | 0.90 (0.50-1.70) | .72 | 60 | |
| Asthma | 12 | 1.70 (0.90-3.20) | .11 | 82 | | 1.40 (1.10-1.80) | .01 | 296 | |
| COPD | 1 | 0.60 (0.10-4.60) | .65 | 18 | | 1.40 (0.80-2.50) | .17 | 61 | |
| Other chronic lung disease | 3 | 1.10 (0.30-3.60) | .86 | 26 | | 1.20 (0.80-1.90) | .39 | 105 | |
| Cancer | 1 | 0.80 (0.10-5.60) | .78 | 14 | | 1.30 (0.70-2.40) | .33 | 51 | |
| History of stroke | 4 | 3.40 (1.20-9.80) | .02 | 14 | | 1.50 (0.80-2.70) | .22 | 47 | |
| Seizures or epilepsy | 4 | 1.00 (0.40-2.70) | .98 | 39 | | 1.20 (0.90-1.80) | .28 | 157 | |
| Intellect./develop. disability | 8 | 1.30 (0.60-2.70) | .49 | 56 | | 1.10 (0.80-1.50) | .46 | 245 | |
| Lack of ability to communicate | 4 | 1.10 (0.40-3.00) | .89 | 27 | | 0.90 (0.60-1.40) | .63 | 145 | |
| Motor weakness or impairment | 14 | 1.40 (0.70-2.40) | .32 | 91 | | 1.10 (0.80-1.30) | .67 | 423 | |
| Spasticity | 2 | 0.50 (0.10-2.10) | .35 | 35 | | 1.20 (0.80-1.70) | .47 | 149 | |
| Dystonia | 3 | 1.10 (0.30-3.50) | .90 | 27 | | 1.20 (0.80-1.90) | .36 | 108 | |
| Reflux | 19 | 2.20 (1.30-3.80) | .004 | 115 | | 1.50 (1.20-1.90) | <.001 | 392 | |
| Delayed gastric emptying | 7 | 1.90 (0.80-4.10) | .13 | 43 | | 1.40 (1.00-2.00) | .06 | 153 | |
| Intestinal motility issues | 5 | 1.00 (0.40-2.40) | .93 | 69 | | 1.80 (1.30-2.40) | <.001 | 201 | |
| Chronic anemia | 2 | 0.80 (0.20-3.40) | .78 | 32 | | 1.70 (1.10-2.60) | .01 | 94 | |
| Inflammatory bowel disease | 1 | 0.30 (0.00-2.40) | .28 | 46 | | 2.10 (1.40-2.90) | <.001 | 113 | |
| Gastric or intestinal polyps | 2 | 1.40 (0.30-5.70) | .66 | 12 | | 1.00 (0.50-1.90) | .94 | 57 | |
| Chronic kidney disease | 2 | 1.20 (0.30-4.90) | .82 | 11 | | 0.80 (0.40-1.50) | .51 | 66 | |
| Chronic liver disease | 0 | 0.60 (0.00-2.90) | .98 | 10 | | 1.20 (0.60-2.30) | .68 | 42 | |
| Glaucoma | 2 | 2.20 (0.50-9.30) | .29 | 13 | | 1.80 (0.90-3.40) | .08 | 36 | |
| Eczema | 2 | 0.50 (0.10-1.90) | .30 | 46 | | 1.40 (1.00-2.00) | .04 | 159 | |
| Other diseases or complications | 17 | 1.40 (0.80-2.50) | .20 | 147 | | 1.60 (1.30-2.00) | <.001 | 494 | |

RDCRN COVID-19 Survey

Completing the survey means that you consent to participate in this research study.

**Please note that all questions are directed to the person with a rare disease: if you are the parent or caregiver, please answer the questions as the person with a rare disease would answer.**

If the survey freezes for any reason, please try refreshing the page.

1. **Please answer the following initial eligibility questions:**

1.1 Do you live in the USA? (y/n, if No -decline)

1.2 Are you:

a. A person with a rare disease?

b. The parent/caregiver for a child under 18-years of age who lives with a rare disease?

c. The parent/caregiver of a person with a rare disease who needs assistance with filling out the survey?

(If none of the above, decline)

1.3 Are you under 90 years of age?

(y/n, If No-decline)

1.4 Please type your disease in the field below: as you type, the system will show you options that may help you find the right diagnosis

1.5 Are you participating in a clinical research project that studies your specific disease, or have done so in the past?

1.6 Can you name the center where the research is (was) conducted? (Y/N/DK, if Yes, list)

1.7 Is (was) the center part of a RDCRN consortium? (Y/N/DK, Drop-down menu of all consortia to help)

**Thank you. Please answer the consent questions below:**

2.1 I agree to provide contact information so that I can participate in a follow-up survey, have my survey answers linked to RDCRN records, and learn about opportunities to participate in research studies conducted by the RDCRN.

2.2 I do not agree to provide my contact information, but would like to participate in the survey. I understand that by not providing the contact information, I cannot participate in a follow-up survey and the RDCRN cannot contact me in the future to let me know about new studies. (Answer to 2.2 can be yes only if answer to 2.1 is No).

1. **CONTACT INFORMATION. (This section will appear only when the participant provides the appropriate consent).**

3.1 Name of person with rare disease:

3.2 Date of birth of person with rare disease

3.3 Name of reporter (person completing the survey)

3.4 Relationship with person: dropdown

1 Self reported

2 Mother

3 Father

4 Step-Mother

5 Step-Father

6 Grandmother

7 Grandfather

8 Aunt

9 Uncle

10 Sister

11 Brother

12 Step-sister

13 Step-brother

14 Legal guardian

15 Other (specify)________________

**How would you like to be contacted? Please provide all information that you are comfortable sharing:**

**3.5 By telephone: area code phone number**

**3.6 By email: email address**

**3.7 By regular mail: street address/apartment number/ city / state / ZIP code**

1. **GENERAL QUESTIONS**
   1. State of residence: (Dropdown containing US states)

4.2 What is your age? (yrs):

4.3 What is your gender? (Dropdown)

Male

Female

Other

Choose not to answer

4.4 What is your race? (Dropdown)

White

Black or African American

American Indian or Native Alaskan

Asian

Native Hawaiian or Pacific Islander

Other

Choose not to answer

4.5 What is your ethnicity? (Dropdown)

Hispanic/Latino

Non-Hispanic or Latino

Unknown

Choose not to answer

4.6 Please check if you had any of the following symptoms before the COVID-19 pandemic began in the USA. Think about your symptoms in January-February 2020. Check all that apply. Did anything change after the beginning of the pandemic (March 2020)? Dropdown containing various symptoms. Columns indicating Before the pandemic (Y/N) and After the beginning of the pandemic (Y/N/less severe/same severity/more severe)

**No symptoms**

**Blood and Bleeding:** blood clots, easy bruising, anemia (low blood cell count), blood in stool, black stools, coughing or vomiting up blood, lip or mouth bleeding, nosebleeds

**Breathing:** cough, wheezing, difficulty breathing, chest pain

**Cardiac:** heart failure, racing heartbeat, arrhythmia

**Digestive:** vomiting, nausea, abdominal pain, bloating, diarrhea, food impaction (getting stuck in the swallowing tube), poor weight gain, weight loss, difficulty swallowing

**Ear, Nose and Throat:** difficulty swallowing, nasal congestion, nasal drainage, ear pain, hearing loss

**Endocrine:** Diabetes, thyroid disease, adrenal insufficiency, hypocalcemia (low calcium in blood), hypoglycemia (low blood sugar), other—specify ___________

**Immune defects:** active infections (bacterial, viral, mycobacterial), abnormal function or amount of white blood cells, recurrent infections (urinary, respiratory, skin)

**Musculoskeletal:** Scoliosis > 30 degrees, Unable to walk more than 50 feet

**Neurological and Behavioral:** headache, migraine, seizures (febrile or other), autism, slurred speech, difficulty swallowing, weakness, tremors, coma, incoordination, intellectual disabilities, cognitive or behavioral impairment, confusion, combativeness, hallucinations

**Kidney:** renal failure, discolored urine

**Skin:** Rash, itching

**Rheumatological:** joint pain or swelling, muscle aches, dry mouth

**Allergies:** anaphylaxis, eczema, hayfever, hives

**Other symptoms:** specify______________________

4.7 Please check if you used any of the treatments before the COVID-19 pandemic began in the USA. Think about the medications you took or treatments you routinely received in January-February 2020. Check all that apply. Did anything change after the beginning of the pandemic (March 2020)? Dropdown containing various medications. Columns indicating Before the pandemic (Y/N) and After the beginning of the pandemic (Y/N/lower dosage/same dosage/higher dosage)

**No medications**

**Antibiotics:** azithromycin (Zithromax), doxycycline, minocycline, other oral antibiotics, inhaled antibiotics (TOBI, Cayston, Colistin), intravenous antibiotics, other _______

**Anticlotting drugs:** warfarin (Coumadin), enoxaparin (Lovenox) injections, other _______

**Biological agents:** Antolimab (AK002), rituximab (Rituxan, MabThera, Truxima), benralizumab (Fasenra), dupilumab (Dupixent), eculizumab (Soliris), mepolizumab (Nucala), reslizumab (Cinqair), bevacizumab (Avastin, Zirabev, MVasi), other _______

**Bone loss prevention drugs:** Bisphosphonates, other_______

**Blood transfusions**

**Breathing drugs:** inhaled albuterol (Ventolin, Proair), inhaled corticosteroids (Pulmicort, Qvar, Flovent, Advair, Symbicort), ipratropium (Atrovent), tiotropium (Spiriva), hypertonic saline (HyperSal), other _______

**Breathing therapies:** home supplemental oxygen, airway clearance techniques (percussion vest, Acapella device, conventional chest physiotherapy), continuous positive airway pressure support (CPAP), bilevel positive airway pressure support (BiPAP), other _______

**Cardiac and blood pressure:** [lisinopril](https://www.medicinenet.com/lisinopril/article.htm) ([Prinivil](https://www.medicinenet.com/lisinopril/article.htm), [Zestril](https://www.medicinenet.com/lisinopril/article.htm), Qbrelis), captopril, enalapril, other _______

**Digestive drugs:** proton pump inhibitors [omeprazole (Prilosec), lansoprazole (Prevacid), pantoprazole (Protonix), rabeprazole (Aciphex), dexlansoprazole (Dexilen)], other _______

**Immunoglobulin infusions** (intravenous or subcutaneous injections), other _______

**Immunosuppressants:** azathioprine (Imuran), mycophenolate (CellCept, Myfortic), tacrolimus (Prograf), Sirolimus (Rapamune), everolimus (Afinitor)

**Steroids:** oral corticosteroids (prednisone), topical swallowed steroids (fluticasone), nonabsorbable oral steroids (budesonide), nasal topical steroids, other _______

**Neurological:** oral anticonvulsants, vigabatrin (Sabril), levetiracetam (Keppra), lamotrigine (Lamictal), clonazepam (Klonopin), clobazam (Onfi), divalproex/valproic acid (Depakote), Riluzole (Rilutek), Edaravone (Radicava, Radicut), dextromethorphan/quinidine (Nuedexta), baclofen, tizanidine (Zanaflex), other _______

**Other drugs:** pyridostigmine (Mestinon), acetazolamide (Diamox), D-galactose, mannose, glycerol phenylbutyrate, sodium phenylbutyrate, sodium benzoate, arginine, citrulline, coenzyme Q10/ubiquinol, oral iron supplement, tranexamic acid, ibuprofen (Advil), naproxen (Aleve), aspirin, other non-steroidal anti-inflammatory drugs, other _______

**Diet:** tube feeding, intravenous nutrition, special diet, elemental diet, food elimination diet, other _______

4.8 Do you have other diseases or complications related to the rare disease? (Yes/No)

If yes, what?

(Dropdown containing the following)

Coronary artery disease, congenital heart disease, heart failure, heterotaxy, hypertension, other heart diseases

Diabetes

Hypothyroidism

Adrenal Insufficiency

Growth Hormone Failure

Asthma, chronic obstructive pulmonary disease (COPD), other chronic lung diseases

Cancer or malignancy. If yes, type _____ Free-text fill-in blanks or Dropdown

History of stroke

Seizures or epilepsy

Intellectual or developmental disabilities

Lack of ability to communicate

Motor weakness or impairment

Spasticity

Dystonia

Chronic bleeding from stomach and/or bowels

Reflux

Delayed gastric emptying

Intestinal motility issues

Chronic anemia

Inflammatory bowel disease (Crohn disease, ulcerative colitis, gluten intolerance, other)

Gastric or intestinal polyps

Chronic kidney disease

Chronic liver disease

Glaucoma

Eczema

History of transplantation. If yes, type _____ Free-text fill-in blanks or Dropdown

Vascular malformations. If yes, specify type and location (lung, liver, brain, stomach, small bowel, large bowel (colon), eye)

Hypereosinophilic Syndrome

Other diseases or complications _______

4.9 Please check if you experienced any of the following symptoms before the COVID-19 pandemic began in the USA. Think about your January-February 2020. Check all that apply.

(Dropdown)

New or increased cough

Fever greater than 100.5 degrees Fahrenheit (38.0 degrees Celsius)

New or increased shortness of breath

Sore throat

Stuffy nose

Runny nose

Chest pain

Sneezing

Wheezing

Headache

Muscles aches

Loss of taste

Loss of smell

Conjunctivitis or pink eye

Confusion

Seizures

Weakness

Other ________ Free text fill-in

4.10 If you experienced COVID-19 symptoms after the beginning of the pandemic in the USA, have you been able to get advice from a health care provider about the opportunity to be tested for COVID-19? (Y/N)

4.11 Do you smoke tobacco cigarettes? Dropdown Yes No Unknown

4.12 Do you currently use other tobacco products? Dropdown Yes No Unknown

4.13 Do you currently use electronic cigarettes or vape? Dropdown Yes No Unknown

4.14 Do you currently smoke marijuana or use THC-containing products? Dropdown Yes No Unknown

4.15 Do you use psychoactive drugs not prescribed by a physician? Dropdown Yes No Unknown

1. **CHANGES AFTER THE BEGINNING OF THE COVID-19 PANDEMIC IN THE USA**
   1. Did you acquire COVID-19? Dropdown (Yes/ No/Don’t know)

(Skip pattern: if No, continue with section 6 below; If Yes, hide Sections 6 and 8 and jump to Section 7, COVID-19 specific questions; If DK, hide Sections 6 and 7 and jump to Section 8)

1. **You answered that you did not acquire the COVID-19 infection.**

6.1 Did you have any of the following symptoms? (Dropdown)

New or increased cough

Fever greater than 100.5 degrees Fahrenheit (38.0 degrees Celsius)

New or increased shortness of breath

Sore throat

Stuffy nose

Runny nose

Chest pain

Sneezing

Wheezing

Headache

Muscles aches

Loss of taste

Loss of smell

Conjunctivitis or pink eye

Confusion

Seizures

Weakness

Other ________ Free text fill-in

No symptoms

6.2 For how many days did you have these symptoms?

6.3 After the beginning of the pandemic in the USA (March 2020), were you able to continue seeing your health care provider?

(Dropdown: Yes, without problems / Yes, but experienced delays in obtaining an appointment/ Yes, but my appointment was done via telemedicine/ No, appointment was put on hold)

If not “Yes without problems,” please describe the issues or difficulties you had ________ Free text fill-in

6.4 After the beginning of the pandemic in the USA (March 2020), were you able to continue your treatment?

(Dropdown: Yes, without problems / Yes, but experienced delays in obtaining treatment/ No, treatment was interrupted)

If not “Yes without problems,” please describe the issues or difficulties you had ________ Free text fill-in

6.5 Were you able to maintain your diet or access food that is necessary for the treatment of your rare disease during the U.S. pandemic?

(Dropdown : Yes, without problems // Yes, but experienced delays/problems //No, supply of needed food was interrupted and my diet suffered from it)

If not “Yes without problems,” please describe the issues or difficulties you had ________ Free text fill-in

6.6 After the beginning of the pandemic in the USA (March 2020), were you able to continue specialized treatment such as occupational therapy or speech therapy?

(Dropdown: Yes, without problems / Yes, but experienced delays in obtaining treatment/ No, treatment was interrupted)

If not “Yes without problems,” please describe the issues or difficulties you had ________ Free text fill-in

6.7 Did you experience a medical event for which you would ordinarily be hospitalized, but because of COVID-19 you were managed without hospitalization? Dropdown Yes No Unknown

If yes, Please explain: ________ Free text fill-in

6.8 Have stay-at-home orders in your area affected your mood or behavior in a way that requires medical attention?

Dropdown Yes No Unknown

If yes, Please explain: ________ Free text fill-in

6.9 Have you or members of your family sought professional support coping with stress or anxiety as a consequence of the COVID-19 pandemic? (Y/N)

6.10 For the parent/caregiver: Did the person you are reporting on die? (Dropdown :Yes/ No )

If yes, please report the cause of death (text)

**Thank you for participating in this important interview. We will post results of this survey as people like you respond, find aggregate survey results on the website of the RDCRN: RDCRN.ORG**

6.11 Please add below any additional comments or concerns you may have.

Free text_________________

**STOP INTERVIEW FOR THOSE WHO ANSWERED THAT THEY DID NOT ACQUIRE COVID-19.**

1. **RESUME HERE INTERVIEW FOR THOSE WHO ANSWERED THAT THEY ACQUIRED COVID-19.**

**You answered that you acquired the COVID-19 infection.**

7.1 When were you diagnosed with COVID-19? Mo/year

7.2 How was the diagnosis of COVID-19 made? Dropdown Specific testing Symptoms Exposure to COVID-19

7.3 What symptoms did you have? Dropdown

New or increased cough

Fever greater than 100.5 degrees Fahrenheit (38.0 degrees Celsius)

New or increased shortness of breath

Sore throat

Stuffy nose

Runny nose

Chest pain

Sneezing

Wheezing

Headache

Muscles aches

Abdominal pain

Vomiting

Diarrhea

Loss of taste

Loss of smell

Conjunctivitis or pink eye

Confusion

Seizures

Weakness

Other ________ Free text fill-in

No symptoms

7.4 For how long did you have symptoms due to COVID-19? (approximate N of days)

7.5 At the time of completing this survey, have your COVID-19 symptoms resolved? Dropdown Yes No Never had symptoms

7.6 Did your rare disease complicate COVID-19? Dropdown Yes No Unknown

If yes, how? ________ Free text fill-in

7.7 Did you experience any worsening of symptoms of your rare disease as a result of COVID-19? Y/N

7.8 After the diagnosis of COVID-19, were you able to continue seeing your health care provider?

(Dropdown: Yes, without problems / Yes, but experienced delays in obtaining an appointment/ Yes, but my appointment was done in telemedicine/ No, appointment was put on hold)

If not “Yes without problems,” please describe the issues or difficulties you had ________ Free text fill-in

7.9 After the diagnosis of COVID-19, were you able to continue your treatment?

(Dropdown: Yes, without problems/ Yes, but experienced delays in obtaining treatment/ No, treatment was interrupted)

If not “Yes without problems,” please describe the issues or difficulties you had ________ Free text fill-in

7.10 After the diagnosis of COVID-19, were you able to maintain your diet or access food that is necessary for the treatment of your rare disease during the pandemic?

(Dropdown: Yes, without problems// Yes, but experienced delays/problems// No, supply of needed food was interrupted and my diet suffered from it )

If not Yes without problem, Please tell us about your complaints ________ Free text fill-in

7.11 After the diagnosis of COVID-19, were you able to continue specialized treatment such as occupational therapy or speech therapy?

(Dropdown: Yes, without problems / Yes, but experienced delays in obtaining treatment/ No, treatment was interrupted)

If not “Yes without problems,” please describe the issues or difficulties you had ________ Free text fill-in

7.12 After the diagnosis of COVID-19, did you experience a medical event for which you would ordinarily be hospitalized, but because of COVID-19 you were managed without hospitalization? Dropdown Yes No Unknown

If yes, how? ________ Free text fill-in

7.13 Have stay-at-home orders in your area affected your mood or behavior in a way that requires medical attention?

Dropdown Yes No Unknown

7.14 Have you or members of your family sought professional support coping with stress or anxiety as a consequence of the COVID-19 pandemic? (Y/N)

7.15 Did you receive investigational drugs to treat COVID-19 or participate in a clinical trial? Dropdown Yes No Unknown

If yes, were you treated with: Dropdown

Chloroquine

Hydroxychloroquine

Oseltamivir (Tamiflu)

Remdesivir

Lopinavir-ritonavir

Azithromycin (specifically for COVID-19)

Oral or intravenous corticosteroids (specifically for COVID-19)

Other medication: specify______________

7.16 Were you seen in an emergency department or urgent care center? Dropdown Yes No Unknown

7.17 Were you hospitalized? Dropdown Yes No Unknown

7.18 Did you require supplemental oxygen? Dropdown Yes No Unknown

7.19 Did you require intubation and mechanical ventilation? Dropdown Yes No Unknown

7.20 For the parent/caregiver: Did the person you are reporting on die? (Yes/ No)

If yes, please report the cause of death (text)

**Thank you for participating in this important interview. We will post results of this survey as people like you respond, find aggregate survey results on the website of the RDCRN: RDCRN.ORG**

7.21 Please add below any additional comments or concerns you may have.

Free text_________________

**STOP INTERVIEW FOR THOSE WHO ANSWERED THAT THEY ACQUIRED COVID-19.**

**8. RESUME HERE INTERVIEW FOR THOSE WHO ANSWERED THAT THEY DID NOT KNOW IF THEY HAD COVID-19.**

**You answered that you do not know if you acquired COVID-19 infection.**

8.1 Were you tested for COVID-19? Yes No

If yes to 8.1: What was the test result? Positive/Negative/Inconclusive/Don’t Know

8.2 Were you exposed to someone who had COVID-19? Y/N/DK

8.3 Did you have symptoms related to COVID-19? Y/N

If answered Yes to 8.3 What symptoms did you have? Dropdown

New or increased cough

Fever greater than 100.5 degrees Fahrenheit (38.0 degrees Celsius)

New or increased shortness of breath

Sore throat

Stuffy nose

Runny nose

Chest pain

Sneezing

Wheezing

Headache

Muscles aches

Abdominal pain

Vomiting

Diarrhea

Loss of taste

Loss of smell

Conjunctivitis or pink eye

Confusion

Seizures

Weakness

Other ________ Free text fill-in

8.4 For how long did you have these symptoms? (approximate N of days)

8.5 At the time of completing this survey, have your symptoms resolved? Dropdown Yes No Never had symptoms

8.6 Did your rare disease complicate this illness? Yes No Unknown

If yes, how? ________ Free text fill-in

8.7 Did you experience any worsening of symptoms of your rare disease as a result of this illness?

Dropdown list of symptoms as for general question – check all that apply

8.8 After the beginning of the pandemic in the USA (March 2020), were you able to continue seeing your health care provider?

(Dropdown: Yes, without problems / Yes, but experienced delays in obtaining an appointment/ Yes, but my appointment was done in telemedicine/ No, appointment was put on hold)

If not “Yes without problems,” please describe the issues or difficulties you had ________ Free text fill-in

8.9 After the beginning of the pandemic in the USA (March 2020), were you able to continue your treatment?

(Dropdown: Yes, without problems/ Yes, but experienced delays in obtaining treatment/ No, treatment was interrupted)

If not Yes without problem, Please tell us about your complaints ________ Free text fill-in

8.10 After the beginning of the pandemic in the USA (March 2020), were you able to maintain your diet or access food that is necessary for the treatment of your rare disease?

(Dropdown: Yes, without problems// Yes, but experienced delays/problems// No, supply of needed food was interrupted and my diet suffered from it )

If not Yes without problem, Please tell us about your complaints ________ Free text fill-in

8.11 After the beginning of the pandemic in the USA (March 2020), were you able to continue specialized treatment such as occupational therapy or speech therapy?

(Dropdown: Yes, without problems / Yes, but experienced delays in obtaining treatment/ No, treatment was interrupted)

If not “Yes without problems,” please describe the issues or difficulties you had ________ Free text fill-in

8.12 After the beginning of the pandemic in the USA (March 2020), did you experience a medical event for which you would ordinarily be hospitalized, but because of COVID-19 you were managed without hospitalization?

Dropdown Yes No Unknown

If yes, Please explain: ________ Free text fill-in

8.13 Have stay-at-home orders in your area affected your mood or behavior in a way that requires medical attention?

Dropdown Yes No Unknown

If yes, Please explain: ________ Free text fill-in

8.14 Have you or members of your family sought professional support coping with stress or anxiety as a consequence of the COVID-19 pandemic? (Y/N)

8.15 For the parent/caregiver: Did the person you are reporting on pass away? (Dropdown :Yes/ No )

If yes, please report the cause of death (text)

**Thank you for participating in this important interview. We will post results of this survey as people like you respond, find aggregate survey results on the website of the RDCRN: RDCRN.ORG**

8.16 Please add below any additional comments or concerns you may have.

Free text_________________

**STOP INTERVIEW FOR THOSE WHO ANSWERED THAT THEY DID NOT KNOW IF THEY HAD COVID-19**
